# The genome sequence of *Triticum turgidum* subsp. *durum* (Desf.) Husnot accession Hoh-501 (Poales: Poaceae)

**DOI:** 10.12688/wellcomeopenres.28084.1

**Published:** 2026-10-09

**Authors:** James Cockram, John Connell, Richard Horsnell, Philip M. Howell, Fiona J. Leigh, Lawrence Percival-Alwyn, Tally I. C. Wright, AEGIS Consortium, Wellcome Sanger Institute Tree of Life Management, Samples and Laboratory team, Wellcome Sanger Institute Scientific Operations: Sequencing Operations, Wellcome Sanger Institute Tree of Life Core Informatics team, Tree of Life Core Informatics collective

**Affiliations:** 1 Niab, Histon, Cambridge, England, CB24 9AT, UK

**Keywords:** Triticum turgidum, Rivet Wheat, genome sequence, chromosomal, Poales

## Abstract

We present a genome assembly of *Triticum turgidum* subsp. *durum* accession ‘Hoh-501’ (allotetraploid, 2 *n* = 4 *x* = 28, AABB; Durum wheat; Streptophyta; Magnoliopsida; Poales; Poaceae). The assembly consists of two haplotypes with total lengths of 10,265.29 megabases and 10,288.95 megabases. Most of the haplotype 1 assembly (99.23%) was scaffolded into 14 chromosomal pseudomolecules. Haplotype 2 was assembled to scaffold level. The mitochondrial genome assembly has a length of 459.37 kilobases and the plastid genome assembly has a length of 135.95 kilobases. This assembly was generated as part of the Ancient Environmental Genomics Initiative for Sustainability (AEGIS) project.

## Species taxonomy

Eukaryota; Viridiplantae; Streptophyta; Streptophytina; Embryophyta; Tracheophyta; Euphyllophyta; Spermatophyta; Magnoliopsida; Mesangiospermae; Liliopsida; Petrosaviidae; commelinids; Poales; Poaceae; BOP clade; Pooideae; Triticodae; Triticeae; Triticinae; *Triticum*; *Triticum turgidum* L. (NCBI:txid4571)

## Background

*Triticum turgidum* subsp. *durum* (Desf.) Husnot, commonly known as durum or pasta wheat, is an allotetraploid species within the Triticeae tribe of the Poaceae family (2 *n* = 4 *x* = 28 chromosomes, AABB genome). It is an annual crop producing approximately 4 to 8 culms (stems) per plant, typically 60 to 150 cm in height. The inflorescence, commonly termed an ‘ear’, consists of spikelets arranged along a central rachis, with around 2 to 4 fertile florets per spikelet. Each floret has an acute lemma apex terminating in an awn of 80 to 150 mm (Figure 1). At maturity, the rachis is non-brittle and the glumes do not strongly adhere to the grain.

Today, durum wheat is grown predominantly for the production of pasta, couscous and speciality breads. It is cultivated across ~17 million hectares worldwide with an annual global production of ~35 million tonnes ( De Vita & Taranto, 2019; Xynias *et al.* , 2020). While the European Union produces the most durum wheat (~9 million tonnes), Canada is the largest producer by individual country (~7 million tonnes), followed by Italy and Turkey ( Agriculture and Agri-Food Canada (AAFC), ; EUROSTAT, ; Xynias *et al.* , 2020). Durum wheat is a relatively drought-adapted crop ( Monneveux *et al.*, 2012) and is commonly grown in semi-arid regions. In the Mediterranean it is typically cultivated as a winter-sown crop, allowing plants to establish and develop over the wetter winter months, with harvest in the early summer. However, in production zones with low winter temperatures such as central Europe and North America, it is grown in the spring and harvested in autumn to avoid cold damage ( Grosse-Heilmann *et al.*, 2024).

Durum wheat was derived from tetraploid emmer wheat via human selection within Neolithic farming systems ( Salamini *et al.* , 2002). It is thought that durum evolved directly from cultivated emmer wheat in the Fertile Crescent (e.g. Özkan *et al.* , 2011; Zohary *et al.*, 2012), although selection from wild emmer wheat has not been definitively excluded ( Oliveira *et al.* , 2012). In common with cultivated emmer wheat, durum has a non-brittle rachis, a domestication trait which prevents ear shattering at maturity and thereby increases harvestable grain. However, while wild and cultivated emmer wheat have hulled grain (in which the glumes remain tightly attached to the grain at maturity, necessitating dehusking prior to consumption), durum wheat has naked, free-threshing grain. Human selection has driven changes in additional durum traits compared to emmer, including increased kernel number per spikelet ( Kiros *et al.* , 2024), altered grain quality and disease resistance. Owing to its evolutionary role as the donor of the AB subgenome of hexaploid bread wheat ( *T. aestivum* L., 2 *n* = 6 *x* = 42, AABBDD genome), the *T. turgidum* gene pool is also an important source of genetic variation for bread wheat improvement ( Levy & Feldman, 2022). This diversity can be accessed either through direct inter-specific crossing (e.g. Horsnell *et al.* , 2024) or via the creation of synthetic hexaploid wheat (SHW), in which tetraploid wheat is crossed with the diploid wild wheat species *Aegilops tauschii* Coss. (2 *n* = 2 *x* = 14, DD genome) followed by embryo rescue and chromosome doubling ( Gaurav *et al.* , 2022).

A reference genome for the Italian durum wheat cultivar ‘Svevo’ spans 10.45 Gb and has a scaffold N50 of 723 Mb ( Maccaferri *et al.* , 2019). More recently, chromosome-scale assemblies have also been released for the U.S. cultivar ‘Langdon’ ( Chen *et al.* , 2025) and the Portuguese durum landrace ‘PI 192051’ ( Yang *et al.* , 2025). Here we present a chromosome-level genome assembly for *Triticum turgidum* subsp. *durum* accession ‘Hoh-501’ (pedigree: ‘Eurodur’ x ‘Charkovskaja32’). Originally developed by the University of Hohenheim, Germany, ‘Hoh-501’ is a winter-type pre-breeding line that has been used as the AB genome donor for >45 synthetic hexaploid wheat accessions generated at Niab, United Kingdom ( Gaurav *et al.* , 2022; Wright *et al.*, 2024).

**Figure 1.**
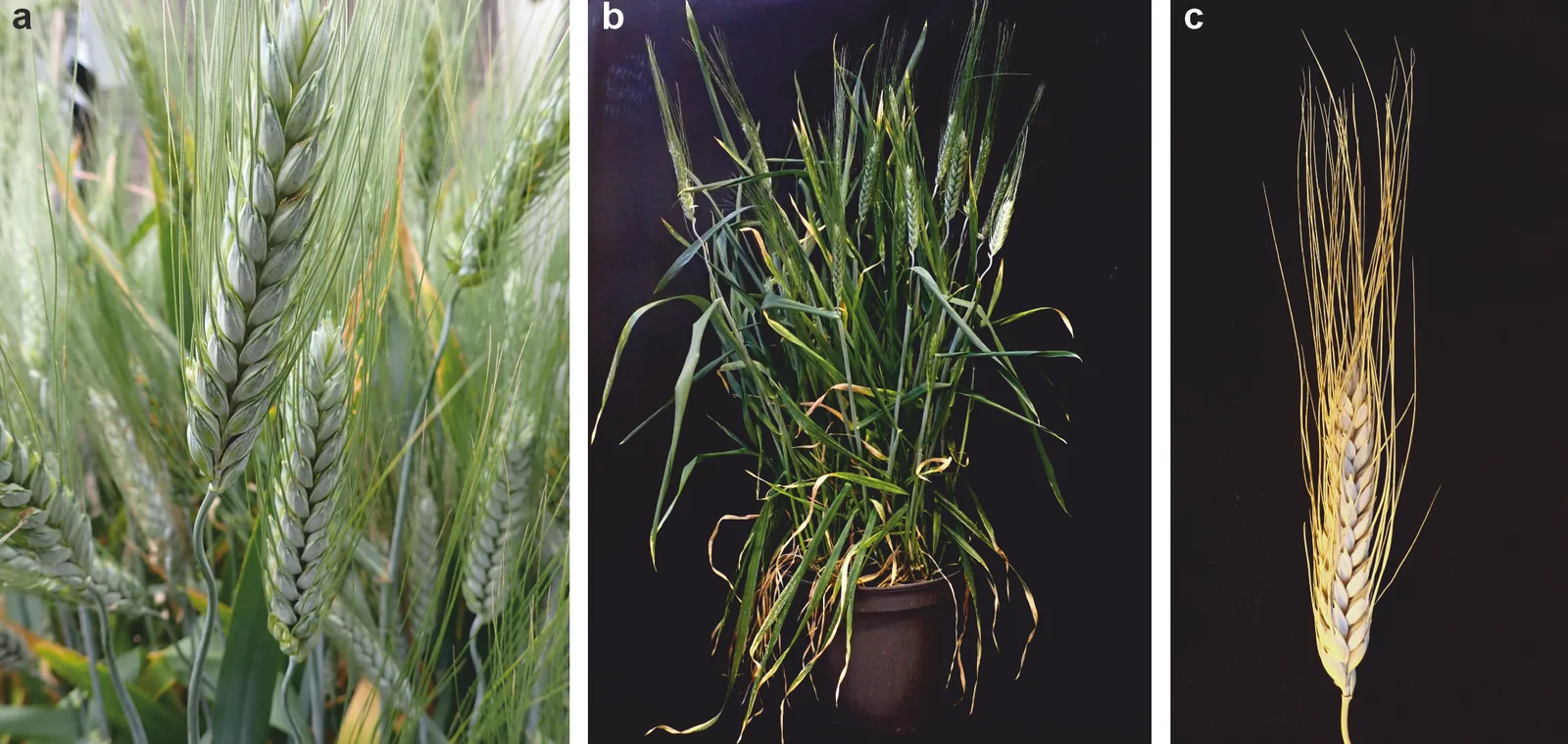
Example images of durum wheat ( *Triticum turgidum* subsp. *durum*) accession ‘Hoh-501’ (a) Ears of an outdoor pot-grown plant at grain-filling stage, June 2025, Cambridge, United Kingdom. (b) Glasshouse-grown individual plants at anthesis, February 2026. (c) An ear at full maturity.

## Methods

### Sample acquisition

A specimen of *T. turgidum* (specimen ID SAN20002194, ToLID lpTriTurg1; Figure 1) was used for genome sequencing. Seedling tissue was collected on 5 January 2024 from plants cultivated in the glasshouse at Niab, Cambridgeshire, UK. The specimen was collected by James Cockram. The lpTriTurg1 specimen was also used for Hi-C and RNA sequencing.

### Nucleic acid extraction

High molecular weight (HMW) DNA extraction protocols developed by the Tree of Life Core Laboratory at the Wellcome Sanger Institute (WSI) are available on protocols.io ( Howard *et al.*, 2025). Tissue from the lpTriTurg1 sample was weighed and triaged to select the appropriate extraction workflow. HMW DNA was prepared for three PacBio HiFi libraries: 6771STDY15062977, 6771STDY15062978 and 6771STDY15062979. The tissue input, HMW DNA extraction, downstream fragmentation and clean-up, and post-shearing quality-control measurements for each PacBio library are summarised in Table 1.

The concentration of the sheared and purified DNA was assessed with a NanoDrop spectrophotometer and a Qubit Fluorometer using the Qubit dsDNA High Sensitivity Assay kit. Fragment size distribution was evaluated by running the sample on the Femto Pulse system.

**Table 1.** DNA extraction and post-extraction processing for each PacBio library used for *T. turgidum*.

**PacBio library ID**	**Tissue preparation**	**HMW DNA extraction**	**Fragmentation and clean-up**	**Post-shearing QC**
6771STDY15062977	115.0 mg seedling; cryogenic bead beating	Automated Plant Organic Extraction	Fragmentation: Megaruptor®3 for LI PacBio; clean-up: Automated SPRI	Qubit 103.0 ng/μL; fragment size 21.4 kb
6771STDY15062978	120.0 mg seedling; cryogenic bead beating	Automated Plant Organic Extraction	Fragmentation: Megaruptor®3 for LI PacBio; clean-up: Automated SPRI	Qubit 104.0 ng/μL; fragment size 19.1 kb
6771STDY15062979	115.0 mg seedling; cryogenic bead beating	Automated Plant Organic Extraction	Fragmentation: Megaruptor®3 for LI PacBio; clean-up: Automated SPRI	Qubit 101.0 ng/μL; fragment size 16.5 kb

RNA was extracted from seedling tissue of lpTriTurg1 in the Tree of Life Laboratory at the WSI using the RNA Extraction: Automated MagMax™ *mir*Vana protocol. The RNA concentration was assessed with a NanoDrop spectrophotometer and a Qubit Fluorometer using the Qubit RNA Broad-Range Assay kit. RNA integrity was assessed using the Agilent RNA 6000 Pico Kit and Eukaryotic Total RNA assay.

### PacBio HiFi library preparation and sequencing

Library preparation and sequencing took place in the WSI Scientific Operations core. Libraries 6771STDY15062977, 6771STDY15062978 and 6771STDY15062979 were prepared with the SMRTbell Prep Kit 3.0 (Pacific Biosciences) according to the manufacturer’s instructions. The kit includes reagents for end repair/A-tailing, adapter ligation, post-ligation SMRTbell bead clean-up, and nuclease treatment. Size selection and clean-up were performed using diluted AMPure PB beads (Pacific Biosciences). DNA concentration was quantified with a Qubit Fluorometer v4.0 (Thermo Fisher Scientific) and the Qubit 1X dsDNA HS assay kit. Final library fragment size was assessed with the Agilent Femto Pulse Automated Pulsed Field CE Instrument (Agilent Technologies) using the gDNA 55 kb BAC analysis kit.

The libraries were sequenced in six runs on a Revio instrument (Pacific Biosciences, California, USA). Prepared libraries with a molarity greater than 2 nM were normalised to 2 nM, and 15 μL was used for making complexes. For libraries with a molarity below 2 nM, the available 10 μL library volume was used without normalisation. Primers were annealed and polymerases were bound to generate circularised complexes, following the manufacturer’s instructions. Complexes were purified using 1.2× SMRTbell beads, diluted to the Revio loading concentration of 200 to 300 pM, and spiked with a Revio sequencing internal control. Sequencing used Revio 25M SMRT cells. SMRT Link software (Pacific Biosciences), a web-based workflow manager, was used to configure and monitor the runs and to carry out primary and secondary data analysis.

### Hi-C

***Sample preparation and crosslinking***

Hi-C data were generated from the lpTriTurg1 seedling sample using the Arima-HiC v2 kit (Arima Genomics). Tissue was finely ground using the Covaris cryoPREP Dry Pulverizer (Covaris), and then subjected to nuclei isolation. Nuclei were isolated using a modified protocol based on the Qiagen QProteome Cell Compartment Kit (Qiagen), in which only the Lysis and CE2 buffers were used, with QIAshredder spin columns. After isolation, nuclei were fixed using formaldehyde to a final concentration of 2% to crosslink the DNA. The crosslinked DNA was then digested and biotinylated according to the manufacturer’s instructions. A clean-up step was performed with SPRIselect beads before library preparation. DNA concentration was quantified using the Qubit Fluorometer v4.0 (Thermo Fisher Scientific) and the Qubit HS Assay Kit, following the manufacturer’s instructions.

***Hi-C library preparation and sequencing***

Biotinylated DNA constructs were fragmented using a Covaris E220 sonicator and size-selected to 400 to 600 bp using SPRISelect beads. DNA was enriched with Arima-HiC v2 kit Enrichment beads. End repair, A-tailing, and adapter ligation were carried out with the NEBNext Ultra II DNA Library Prep Kit (New England Biolabs), following a modified protocol where library preparation occurs while DNA remains bound to the Enrichment beads. Libraries were amplified with KAPA HiFi HotStart mix and a custom Unique Dual Index (UDI) barcode set (Integrated DNA Technologies). Depending on sample concentration and biotinylation percentage determined at the crosslinking stage, libraries were amplified with 10 to 16 PCR cycles. Post-PCR clean-up was performed with SPRISelect beads. Libraries were quantified using the AccuClear Ultra High Sensitivity dsDNA Standards Assay Kit (Biotium) and a FLUOstar Omega plate reader (BMG Labtech).

Prior to sequencing, libraries were normalised to 10 ng/μL. Normalised libraries were quantified again to create equimolar and/or weighted 2.8 nM pools. Pool concentrations were checked using the Agilent 4200 TapeStation (Agilent) with High Sensitivity D500 reagents before sequencing. Libraries were sequenced on the Illumina NovaSeq X, generating paired-end 150 bp reads.

### RNA library preparation and sequencing

Libraries were prepared with the NEBNext® Ultra II Directional RNA Library Prep Kit for Illumina (New England Biolabs), following the manufacturer’s instructions. Poly(A) mRNA was isolated from 100 ng of total RNA using oligo(dT) beads, converted to cDNA, and uniquely indexed; 14 PCR cycles were performed. Libraries were prepared with an intended average fragment size of 100 to 300 bp. Libraries were quantified, normalised, and pooled equimolarly to a final concentration of 2.8 nM before dilution to 150 pM for loading. Sequencing was carried out on the Illumina NovaSeq X, generating paired-end reads.

### Genome assembly

Prior to assembly of the PacBio HiFi reads, a database of *k*-mer counts ( *k* = 31) was generated from the filtered reads using FastK. The *k*-mer frequency distribution was analysed with GenomeScope (version 2.1.0) ( Ranallo-Benavidez *et al.*, 2020), providing fitted point estimates of genome size, heterozygosity and repeat content.

The HiFi reads were assembled using Hifiasm in Hi-C phasing mode ( Cheng *et al.*, 2021, 2022), producing two haplotypes. Hi-C reads ( Rao *et al.* , 2014) were mapped to contigs using bwa-mem2 ( Vasimuddin *et al.*, 2019). Contigs were further scaffolded with Hi-C data in YaHS ( Zhou *et al.*, 2023), using the --break option for handling potential misassemblies. The scaffolded assemblies were evaluated using Gfastats ( Formenti *et al.* , 2022), BUSCO ( Manni *et al.*, 2021) and MerquryFK ( Rhie *et al.*, 2020). The organelle genomes were assembled using OATK ( Zhou *et al.*, 2025). The comparator for the mitochondrial assembly was the mitochondrial genome of *Triticum aestivum* (NC_036024.1).

### Assembly curation

The assembly was decontaminated using the Assembly Screen for Cobionts and Contaminants ( ASCC) pipeline. The flat files and maps for curation were generated with TreeVal. Manual curation was conducted primarily in PretextView and HiGlass ( Kerpedjiev *et al.* , 2018). Scaffolds were visually inspected and corrected as described by Howe *et al*. (2021). The two haplotype assemblies were combined for curation. Manual corrections included 66 breaks and 101 joins. This reduced the scaffold count by 2.3% and increased the scaffold N50 by 3.3%. The curation process is described at sanger-tol/curation-resources. A Hi-C contact map of the haplotype 1 assembly was generated with PretextSnapshot.

### Assembly quality assessment

The MerquryFK tool ( Rhie *et al.*, 2020) was run in a Singularity container ( Kurtzer *et al.*, 2017) to evaluate *k*-mer completeness and assembly quality for both haplotypes using the *k*-mer database ( *k* = 31) computed prior to genome assembly. The analysis outputs included assembly QV scores and completeness statistics.

The haplotype 1 assembly was assessed using BlobToolKit ( Challis *et al.*, 2020). PacBio reads were mapped to the assembly to derive sequence coverage, and BUSCO ( Manni *et al.*, 2021) was used to assess gene-set completeness. DIAMOND BLASTp searches of extracted BUSCO proteins and DIAMOND BLASTx searches of assembly sequences against UniProt Reference Proteomes ( Buchfink *et al.*, 2021), followed by BLASTn searches of sequences without DIAMOND BLASTx hits against the NCBI nt database ( Altschul *et al.*, 1990), supported taxonomic assignment. Coverage, sequence composition, taxonomic assignment and BUSCO results were integrated into a BlobDir for interactive inspection and visualisation. The snail and blob plots were rendered locally from this hosted BlobDir using BlobTk v0.8.3. The scaffold-length radial scale function for the snail plot was set to linear ( Challis & Blaxter, 2026). The blob plot used circular markers with area proportional to scaffold length ( Challis *et al.*, 2020).

## Genome sequence report

### Sequence data

The genome of a specimen of *T. turgidum* was sequenced using Pacific Biosciences single-molecule HiFi long reads, generating 426.87 Gb (gigabases) from 35.03 million reads. We assembled the genome from these reads. GenomeScope analysis using a *p* = 4 model gave a *k*-mer-based haploid genome size estimate of 5,362.73 Mb, with heterozygosity of 6.44% and repeat content of 72.41%; the full-model fit was 88.24% (Figure 2). This estimate guided expectations for the assembly. The sequencing data provided a reported 39× coverage of the genome. Hi-C sequencing produced 2,330.55 Gb from 7,717.05 million read pairs, which were used to scaffold the haplotype 1 assembly. RNA sequencing data were also generated and are available in public sequence repositories. Table 2 summarises the specimen and sequencing details.

**Figure 2.**
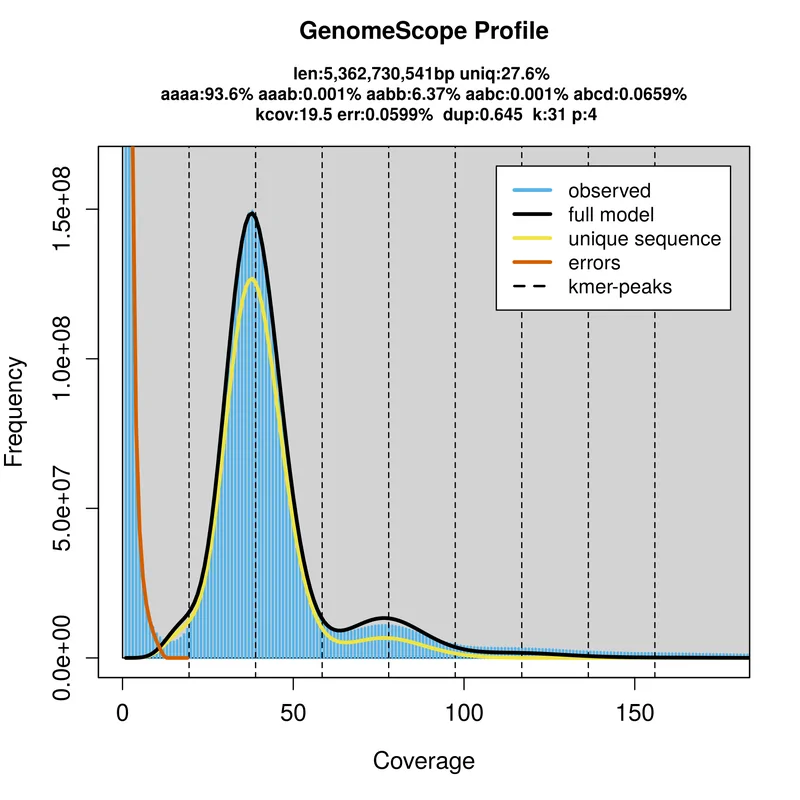
GenomeScope model fitted to the *k*-mer frequency distribution. The plot shows the observed and modelled *k*-mer spectra used to estimate genome size, heterozygosity and repeat content from unassembled sequencing reads.

**Table 2.** Specimen and sequencing data for *T. turgidum* (BioProject PRJEB85737)

**Platform**	**PacBio HiFi**	**Hi-C**	**RNA-seq**
**ToLID**	lpTriTurg1	lpTriTurg1	lpTriTurg1
**Specimen ID**	SAN20002194	SAN20002194	SAN20002194
**BioSample (source individual)**	SAMEA115133505	SAMEA115133505	SAMEA115133505
**BioSample (tissue)**	SAMEA115133611	SAMEA115133611; SAMEA115133614	SAMEA115133614; SAMEA115133611
**Tissue**	seedling	seedling	seedling
**Instrument**	Revio	Illumina NovaSeq X	Illumina NovaSeq X
**Library ID & run accession(s)**	6771STDY15062977: ERR14375745, ERR14375748; 6771STDY15062978: ERR14375746, ERR14375749; 6771STDY15062979: ERR14375747, ERR14375750	ERR14379172; ERR14379173	ERR14379174; ERR14379175
**Read count total**	35.03 million reads	7,717.05 million read pairs	73.98 million read pairs
**Base count total**	426.87 Gb	2,330.55 Gb	22.34 Gb

### Assembly statistics

The genome was assembled into two haplotypes using Hi-C phasing. The haplotype 1 assembly was curated to chromosome level, while haplotype 2 was assembled to scaffold level. The haplotype 1 assembly has a total length of 10,265.29 Mb in 1,086 scaffolds (excluding organelle sequences), with 1,111 gaps, and a scaffold N50 of 739.56 Mb (Table 3). The haplotype 1 assembly has a scaffold auN of 728 Mb.

**Table 3.** Genome assembly statistics for *T. turgidum*

**Genome assembly**	**Haplotype 1**	**Haplotype 2**
**Assembly name**	lpTriTurg1.hap1.1	lpTriTurg1.hap2.1
**Assembly accession**	GCA_965278285.1	GCA_965278335.1
**Assembly level**	chromosome	scaffold
**Span (Mb)**	10,265.29	10,288.95
**Number of chromosomes**	14	-
**Number of contigs**	2,197	1,712
**Contig N50 (Mb)**	31.62	32.99
**Number of scaffolds (excluding organelle sequences)**	1,086	816
**Scaffold N50 (Mb)**	739.56	739.7
**Scaffold auN (Mb)**	728	729
**Longest scaffold length (Mb)**	838.91	841.6
**Organelles**	Mitochondrial genome: 459.37 kb; Plastid genome: 135.95 kb	-

Most of the haplotype 1 assembly sequence (99.23%) was assigned to 14 chromosome-level scaffolds. These chromosome-level scaffolds, confirmed by Hi-C data, are named according to synteny (Figure 3; Table 4).

The mitochondrial genome (length 459.37 kb, OZ256559.1) and plastid genome (length 135.95 kb, OZ256560.1) were also assembled. These sequences are included as contigs in the multifasta file of the haplotype 1 genome submission and as standalone records.

**Figure 3.**
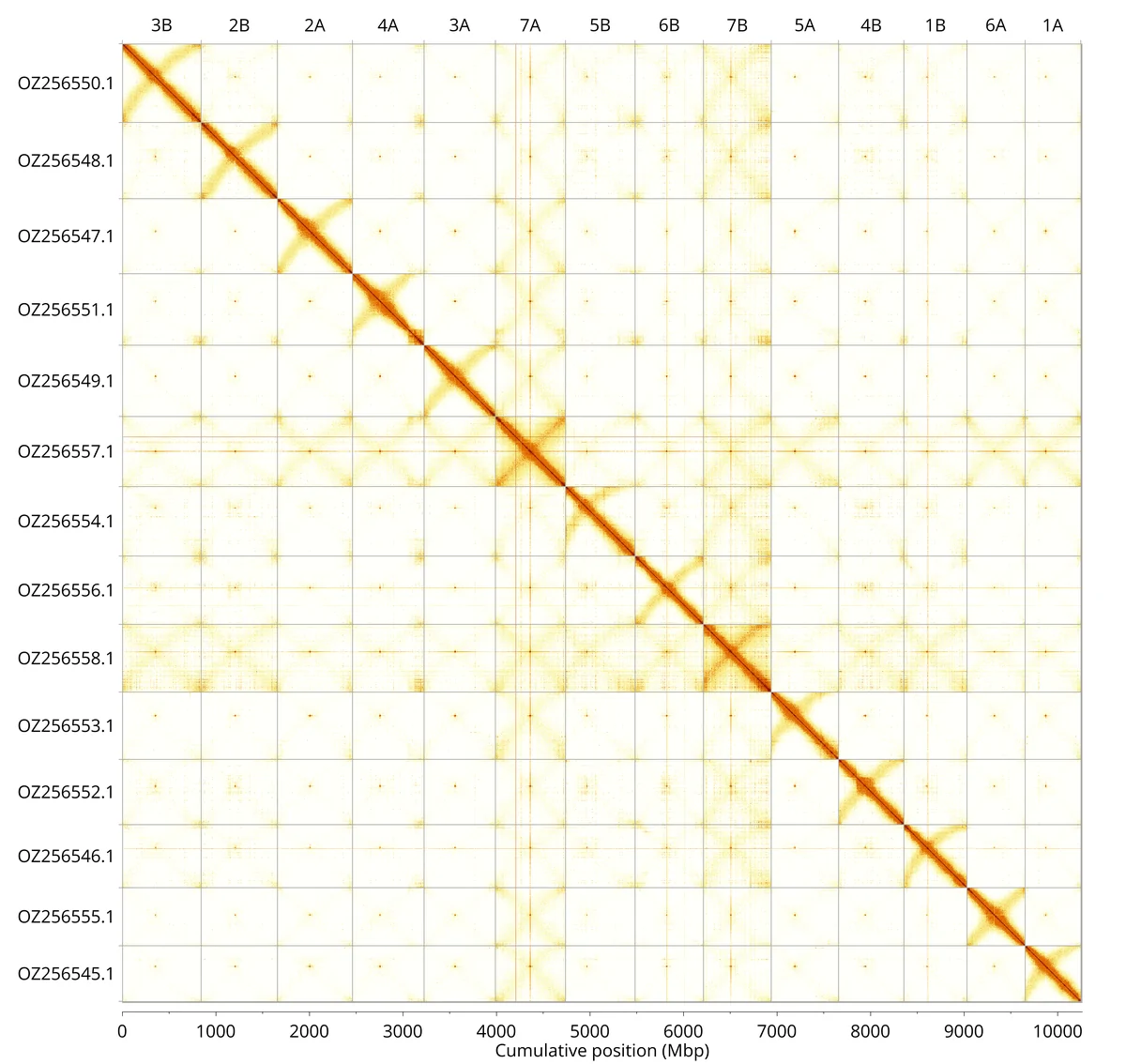
Hi-C contact map of the *T. turgidum* haplotype 1 assembly. Assembled chromosomes are shown in order of size and labelled along the axes, with a megabase scale shown below. The plot was generated using PretextSnapshot.

**Table 4.** Chromosomal pseudomolecules in the haplotype 1 genome assembly of *T. turgidum* lpTriTurg1

**INSDC accession**	**Molecule**	**Length (Mb)**	**GC%**
OZ256545.1	1A	591.86	46
OZ256546.1	1B	671.28	46
OZ256547.1	2A	797.89	46
OZ256548.1	2B	810.73	46
OZ256549.1	3A	757.08	46
OZ256550.1	3B	839.67	46
OZ256551.1	4A	760.43	46
OZ256552.1	4B	694.32	46.50
OZ256553.1	5A	713.81	46
OZ256554.1	5B	739.56	46
OZ256555.1	6A	618.04	46
OZ256556.1	6B	725.50	46.50
OZ256557.1	7A	743.83	46
OZ256558.1	7B	722.09	46

### Assembly quality metrics

For the haplotype 1 assembly, the estimated QV is 68.9, and for the haplotype 2 assembly, 69.7. When the two haplotypes are combined, the assembly achieves an estimated QV of 69.2. The *k*-mer completeness is 97.98% for the haplotype 1 assembly, 97.91% for the haplotype 2 assembly, and 99.11% for the combined haplotypes (Figure 4).

BUSCO analysis using the poales_odb10 reference set ( *n* = 4,896) identified 99.0% of the expected gene set (single = 9.9%, duplicated = 89.1%) in the haplotype 1 assembly. For the haplotype 2 assembly, BUSCO v.5.7.1 analysis identified 99.0% of the expected gene set (single = 10.3%, duplicated = 88.7%).

**Figure 4.**
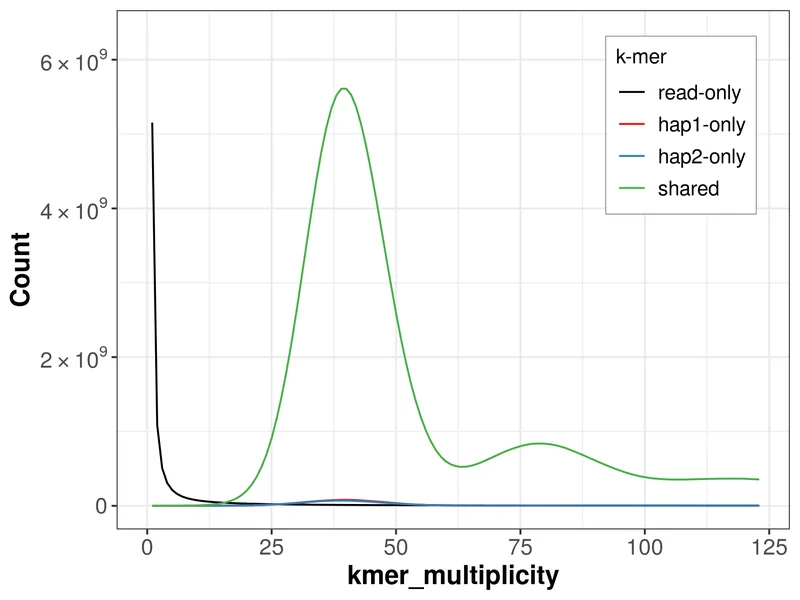
Evaluation of *k*-mer completeness using MerquryFK. This plot illustrates the recovery of *k*‐mers from the original read data in the haplotype 1 assembly, the haplotype 2 assembly and the combined haplotypes. The horizontal axis represents *k*‐mer multiplicity, and the vertical axis shows the number of *k*‐mers. The black curve represents *k*‐mers that appear in the reads but are not assembled, while the remaining curves correspond to *k*‐mers recovered in the assemblies shown.

The snail plot in Figure 5 provides a summary of assembly contiguity, cumulative scaffold count, sequence composition and BUSCO completeness for the haplotype 1 assembly ( Challis & Blaxter, 2026). The relative sizes of the longest scaffold, scaffold N50 and N90, together with the dark-grey scaffold-length distribution, show how much of the assembly is contained in long scaffolds. The outer composition track shows variation in GC, AT and N content, while the central track shows the cumulative scaffold count and the BUSCO inset summarises gene-set completeness.

The blob plot in Figure 6 displays scaffolds from the haplotype 1 assembly by GC proportion and base coverage. Marker area is proportional to scaffold length, and colour indicates taxonomic assignment ( Challis *et al.*, 2020).

**Figure 5.**
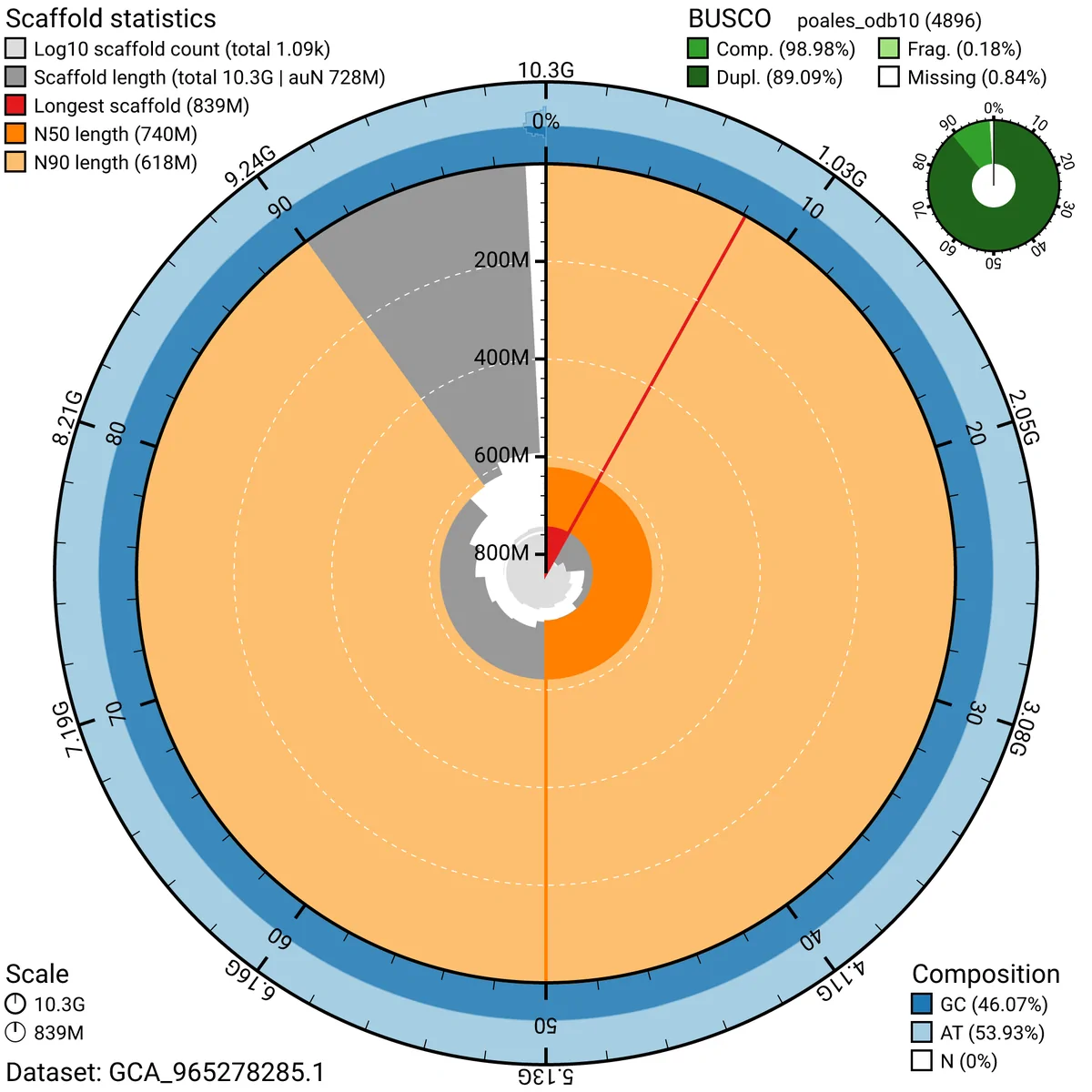
Assembly metrics for lpTriTurg1.hap1.1, the haplotype 1 assembly. The BlobToolKit snail plot provides an overview of assembly metrics and BUSCO gene completeness. The circumference represents the length of the haplotype 1 assembly, and the main plot is divided into 1,000 bins around the circumference. The outermost blue tracks display the distribution of GC, AT, and N percentages across the bins. Scaffolds are arranged clockwise from longest to shortest and are depicted in dark grey. Scaffold length is shown on a linear radial scale, scaled to the longest scaffold. Red shading indicates the longest scaffold, and the deeper orange and pale orange shading represent the N50 and N90 lengths. The summary statistics include scaffold auN, the area under the scaffold Nx curve. The light grey spiral at the centre shows the cumulative scaffold count on a logarithmic scale. A summary of complete, fragmented, duplicated, and missing BUSCO genes in the poales_odb10 set is presented at the top right. The assembly can be explored interactively in the BlobToolKit viewer.

**Figure 6.**
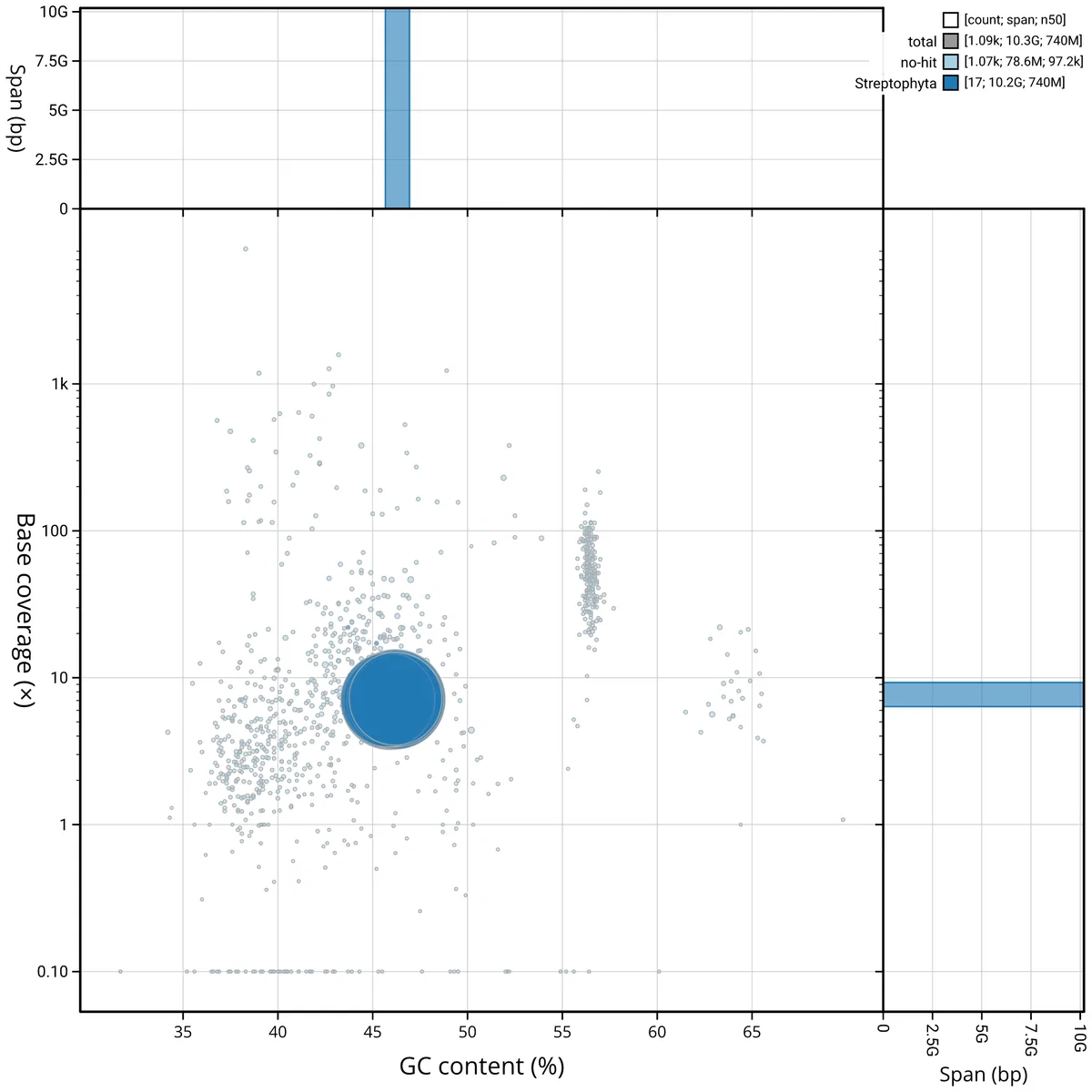
BlobToolKit blob plot for lpTriTurg1.hap1.1, the haplotype 1 assembly. The plot shows base coverage (vertical axis) and GC content (horizontal axis). Circles represent scaffolds; marker area is proportional to scaffold length, and colour indicates taxonomic assignment. The histograms along the axes display the total length of sequences distributed across different levels of coverage and GC content. The assembly can be explored interactively in the BlobToolKit viewer.

Table 5 presents assembly quality metrics alongside the Earth BioGenome Project (EBP) benchmarks described by Howe *et al.*. (2026). The three-part EBP metric calculated for the haplotype 1 assembly is **7.C.Q68**.

**Table 5.** Earth BioGenome Project summary metrics for the *T. turgidum* assembly

**Measure**	**Value**	***Benchmark***
EBP summary (haplotype 1)	7.C.Q68	*6.C.Q40*
Contig N50 length	31.62 Mb	*> 1 Mb*
Scaffold N50 length	739.56 Mb	*= chromosome N50*
Consensus quality (QV)	Haplotype 1: 68.9; haplotype 2: 69.7; combined: 69.2	*≥ 40*
*k*-mer completeness	Haplotype 1: 97.98%; Haplotype 2: 97.91%; combined: 99.11%	*> 90%*
BUSCO	Haplotype 1: C:99.0% [S:9.9%, D:89.1%], F:0.2%, M:0.8%, n:4,896; Haplotype 2: C:99.0% [S:10.3%, D:88.7%], F:0.2%, M:0.9%, n:4,896	*C > 90%*
Percentage of assembly assigned to chromosomes	99.23%	*≥ 90%*

**Notes:** The EBP summary uses log10(Contig N50); chromosome-level (C) or log10(Scaffold N50); Q (Merqury QV). BUSCO v5.7.1 using the poales_odb10 lineage dataset: C=complete; S=single-copy; D=duplicated; F=fragmented; M=missing; n=orthologues. For plants, the BUSCO benchmark uses total completeness (C = S + D) to accommodate biological duplication; this is a plant-specific adaptation of the EBP single-copy benchmark.

### Data availability

European Nucleotide Archive: *Triticum turgidum* (pasta wheat). Accession number PRJEB85737; https://identifiers.org/ena.embl/PRJEB85737. The genome sequence is released openly for reuse. The *T. turgidum* genome sequencing initiative is part of the Sanger Institute Tree of Life Programme ( PRJEB43745) and AEGIS ( PRJEB80366). All raw sequence data and the haplotype assemblies have been deposited in INSDC databases. The genome will be annotated using available RNA-Seq data and presented through the Ensembl pipeline at the European Bioinformatics Institute. Raw data and assembly accession identifiers are reported in Table 2 and Table 3.

Pipelines used for genome assembly at the WSI Tree of Life are available at https://pipelines.tol.sanger.ac.uk/pipelines. Table 6 lists software versions used in this study.

**Table 6.** Software versions and sources

**Software**	**Version**	**Source**
BLAST	2.14.0	https://ftp.ncbi.nlm.nih.gov/blast/executables/blast+/
BlobToolKit	4.4.5	https://github.com/genomehubs/blobtoolkit
BlobTk (hosted BlobDir)	0.5.1	https://github.com/genomehubs/blobtk
BlobTk (snail and blob plot renderer)	0.8.3	https://github.com/genomehubs/blobtk
BUSCO	5.7.1; 6.1.0	https://gitlab.com/ezlab/busco
bwa-mem2	2.2.1	https://github.com/bwa-mem2/bwa-mem2
DIAMOND	2.1.8	https://github.com/bbuchfink/diamond
fasta_windows	0.2.4	https://github.com/tolkit/fasta_windows
FastK	1.2	https://github.com/thegenemyers/FASTK
GenomeScope	2.1.0	https://github.com/tbenavi1/genomescope2.0
Gfastats	1.3.6	https://github.com/vgl-hub/gfastats
Hifiasm	0.19.8-r603	https://github.com/chhylp123/hifiasm
HiGlass	1.13.4	https://github.com/higlass/higlass
MerquryFK	1.1.0-c1	https://github.com/thegenemyers/MERQURY.FK
Minimap2	2.28-r1209	https://github.com/lh3/minimap2
Oatk	0.9	https://github.com/c-zhou/oatk
MultiQC	1.14; 1.17 and 1.18	https://github.com/MultiQC/MultiQC
Nextflow	24.10.4	https://github.com/nextflow-io/nextflow
PretextSnapshot	0.0.6	https://github.com/sanger-tol/PretextSnapshot
PretextView	1.0.3	https://github.com/sanger-tol/PretextView
samtools	1.21	https://github.com/samtools/samtools
sanger-tol/ascc	0.1.0	https://github.com/sanger-tol/ascc
sanger-tol/blobtoolkit	v0.7.1	https://github.com/sanger-tol/blobtoolkit
sanger-tol/curationpretext	1.4.2	https://github.com/sanger-tol/curationpretext
Seqtk	1.4-r122	https://github.com/lh3/seqtk
Singularity	3.9.0	https://github.com/sylabs/singularity
TreeVal	1.2.2	https://github.com/sanger-tol/treeval
YaHS	1.2.2	https://github.com/c-zhou/yahs

### Author information

Contributors represented by collective authorship are listed at the following links:

- Members of the Wellcome Sanger Institute Tree of Life Management, Samples and Laboratory team
- Members of Wellcome Sanger Institute Scientific Operations – Sequencing Operations
- Members of the Wellcome Sanger Institute Tree of Life Core Informatics team
- Members of the Tree of Life Core Informatics collective
- Members of the AEGIS Consortium

### Wellcome Sanger Institute – Legal and Governance

The materials that have contributed to this genome note have been supplied by a Tree of Life collaborator. The Wellcome Sanger Institute employs a process whereby due diligence is carried out proportionate to the nature of the materials themselves, and the circumstances under which they have been/are to be collected and provided for use. The purpose of this is to address and mitigate any potential legal and/or ethical implications of receipt and use of the materials as part of the research project, and to ensure that in doing so we align with best practice wherever possible.

The overarching areas of consideration are:

- Ethical review of provenance and sourcing of the material
- Legality of collection, transfer and use (national and international)

Each transfer of samples is undertaken according to a Research Collaboration Agreement or Material Transfer Agreement entered into by the Tree of Life collaborator, Genome Research Limited (operating as the Wellcome Sanger Institute) and in some circumstances other Tree of Life collaborators.
