# Supplementary material for: The genome sequence of Triticum turgidum subsp. durum (Desf.) Husnot accession Hoh-501 (Poales: Poaceae)
Source: Wellcome Open Res. 2026 Oct 9;11:840. [Version 1] doi: 10.12688/wellcomeopenres.28084.1 (PMC13680841; doi:10.12688/wellcomeopenres.28084.1)
Supplement: Peer review history

# Reviewer report: Reviewer response for version 1

**DOI:** 10.21956/wellcomeopenres.30900.r174453

**Published:** 2026-10-22

**Authors:** Martin Mascher

**Affiliations:** 1 Leibniz Institute of Plant Genetics and Crop Plant Research, Seeland, Germany

The authors present a high-quality genome assembly of the durum wheat accession Hoh-501, which has been used as an AB genome donor for the production of synthetic hexaploid wheat.

 I have a few comments that I would encourage the authors to address.

 1. The GenomeScope analysis reports a genome size of 5.36 Gb and heterozygosity of 6.44%. The genome size estimate is approximately half the assembly size, presumably because it represents the monoploid genome size. This should be clarified. Furthermore, the high heterozygosity estimate seems unlikely to represent true allelic heterozygosity in this wheat line. Differences between the A and B subgenomes may affect the interpretation of the k-mer spectrum. I suggest repeating the analysis with a larger k-mer size, such as 71 or 101, and/or using an alternative genome size estimation tool, such as findGSE (Sun et al., 2018, Bioinformatics). The authors should also clarify the limitations of heterozygosity estimates derived from k-mer spectra in an allotetraploid genome.

 2. The authors report two haplotype assemblies, each approximately 10.3 Gb in size. It would be helpful to clarify how these assemblies should be interpreted in the context of an allotetraploid and presumably largely homozygous wheat line. In particular, is there any information about the degree of allelic divergence or the accuracy of haplotype separation?

 3. The chromosome-level assembly appears well supported by the Hi-C contact map. A comparison with an existing durum wheat reference, such as Svevo or Zavitan, would provide additional evidence for structural accuracy.

 4. The keyword "Rivet Wheat" appears inappropriate for T. turgidum subsp. durum and should be reconsidered.

Are the datasets clearly presented in a usable and accessible format, and the assembly and annotation available in an appropriate subject-specific repository?

Yes

Are sufficient details of the sequencing and extraction, software used, and materials provided to allow replication by others?

Yes

Are the rationale for sequencing the genome and the species significance clearly described?

Yes

Are the protocols appropriate and is the work technically sound?

Yes

Reviewer Expertise:

Plant genomics, barley, wheat, genome sequence assembly

I confirm that I have read this submission and believe that I have an appropriate level of expertise to confirm that it is of an acceptable scientific standard.

# Reviewer report: Reviewer response for version 1

**DOI:** 10.21956/wellcomeopenres.30900.r174450

**Published:** 2026-10-19

**Authors:** Ruslan Kalendar

**Affiliations:** 1 University of Helsinki, Helsinki, Finland

The authors present a genome assembly of allotetraploid durum wheat (2n = 4x = 28, AABB) accession Hoh-501, a winter-type pre-breeding line from the University of Hohenheim that Niab has used as the AB-genome donor for more than 45 synthetic hexaploid wheats. The work follows the Sanger Tree of Life Genome Note format and was produced within the AEGIS project.

 The data comprise 426.87 Gb of PacBio HiFi reads (Revio; three libraries, six runs), 2,330.55 Gb of Hi-C and 22.34 Gb of RNA-seq (NovaSeq X). Assembly used hifiasm in Hi-C phasing mode, YaHS scaffolding and manual curation (66 breaks, 101 joins). In haplotype 1, 99.23% of the sequence is assigned to 14 pseudomolecules; mitochondrial (459.37 kb) and plastid (135.95 kb) genomes were also assembled. The article contains no annotation; this is deferred to the Ensembl pipeline.

 The Sequence data section reports a haploid genome size estimate of 5,362.73 Mb (p = 4 model), yet each haplotype assembly spans 10.27 Gb. The discrepancy is exactly two-fold and is not discussed anywhere. Presumably GenomeScope with p = 4 returns the monoploid (1x) length, i.e. a single subgenome, whereas the gametic AABB complement is 10.7 Gb, consistent with the assembly and with the Svevo genome (10.45 Gb).  

 The same problem affects coverage: 426.87 Gb / 10,265.29 Mb = 41.6×, and 426.87 Gb / 5,362.73 Mb = 79.6×. The reported 39× cannot be reproduced from any pair of numbers in the article (the closest is division by 10.95 Gb, which resembles 2 × the kcov of 19.5 shown in Figure 2).  

 The Hi-C mode of hifiasm is designed for diploids, yet here it is applied to an allotetraploid and to a near-homozygous line. The two resulting "haplotypes" are practically indistinguishable: 10,265.29 vs 10,288.95 Mb, scaffold N50 739.56 vs 739.7 Mb, auN 728 vs 729 Mb, BUSCO 99.0% for both. In Figure 4, the hap1-only and hap2-only curves lie close to zero.

 Table 3 gives a longest scaffold of 838.91 Mb, Table 4 gives chromosome 3B as 839.67 Mb, and Figure 5 shows 839M. The longest scaffold cannot be shorter than a chromosome-level scaffold.

 The 0.76 Mb difference is plausibly explained by one value excluding gaps and the other not. However, the scaffold N50 in Table 3 (739.56 Mb) exactly equals the full length of 5B in Table 4, so the convention is inconsistent even within a single table.

Are the datasets clearly presented in a usable and accessible format, and the assembly and annotation available in an appropriate subject-specific repository?

Yes

Are sufficient details of the sequencing and extraction, software used, and materials provided to allow replication by others?

Yes

Are the rationale for sequencing the genome and the species significance clearly described?

Yes

Are the protocols appropriate and is the work technically sound?

Yes

Reviewer Expertise:

plant genomics; assembly and quality assessment of polyploid genomes

I confirm that I have read this submission and believe that I have an appropriate level of expertise to confirm that it is of an acceptable scientific standard.

# Reviewer report: Reviewer response for version 1

**Published:** 2026-10-09

**Authors:** Automated Benchmarking

**Affiliations:** 1 Wellcome Sanger Institute, Hinxton, UK

As this article is a Genome Note, Automated Benchmarking has been used to determine whether the reported assembly passes four defined checks established by the Earth BioGenome Project: the raw and assembled data are publicly available, the Earth BioGenome Project three-part metric is met, the k-mer completeness exceeds 90%, and the BUSCO score passes the stated threshold. For Triticum turgidum (lpTriTurg1), all four checks passed. Data available: pass. ENA study PRJEB85737, raw-read accessions ERR14375745, ERR14375746, ERR14375747, ERR14375748, ERR14375749, ERR14375750, ERR14379172, ERR14379173, ERR14379174, and ERR14379175, and assembly accessions GCA_965278285.1 and GCA_965278335.1 are public. Earth BioGenome Project three-part metric: pass. Haplotype 1 is reported as 7.C.Q68, meeting the 6.C.Q40 reference standard. k-mer completeness: pass. The combined haplotype assemblies have 99.11% k-mer completeness, exceeding the Earth BioGenome Project target of 90%. BUSCO: pass. Haplotype 1, which is proposed as the reference assembly for this species, has 99.0% complete BUSCOs (9.9% single-copy and 89.1% duplicated). BUSCO software version: 5.7.1; lineage dataset: poales_odb10. It meets the plant-specific target of greater than 90% complete BUSCOs. This decision uses values reported in the Genome Note and public accession records.

Reviewer Expertise:

NA

I confirm that I have read this submission and believe that I have an appropriate level of expertise to confirm that it is of an acceptable scientific standard.
